# Disease reciprocity between gingivitis and obesity

**DOI:** 10.1002/JPER.20-0046

**Published:** 2020-08-06

**Authors:** J. Max Goodson

**Affiliations:** ^1^ The Forsyth Institute Cambridge MA

**Keywords:** collagen, gingivitis, hydroxyproline, metabolomics, obesity

## Abstract

**Background:**

Many diseases seem to affect each other. This is particularly true of periodontal diseases that relate to many systemic diseases. For this reason, this study investigated the relationship between obesity and gingivitis in children by focusing on plasma and salivary metabolomic biochemicals.

**Methods:**

Whole saliva and plasma samples were taken from each of sixty‐eight 11‐year‐old children afflicted by different degrees of both gingivitis and obesity. Gingivitis was evaluated as the percent of sites considered erythematous. Obesity was determined by waist circumference. Untargeted metabolomic analysis defined 29 biochemicals significantly correlated between saliva and plasma, which included the collagen breakdown amino acid hydroxyproline (Hyp). Two‐sided *t*‐tests and regression analysis were performed to compare these data from children with obesity alone, gingivitis alone, both, and neither.

**Results:**

Obese children exhibited signs of increased collagen turnover by being taller (14.4 cm) and having more permanent teeth (5.7). Analysis indicated a significant impact of obesity on gingivitis. Children with both diseases had 41.02% of gingival sites red whereas children with only obesity had 5.2% and children with only gingivitis had 19.16%. Hyp was increased in saliva by the combined presence of both diseases. The effects of gingivitis on obesity were in the same direction but generally not statistically significant.

**Conclusion:**

Obesity clearly augments gingivitis. Data suggest that interaction between gingivitis and obesity may exhibit disease reciprocity in which activated neutrophils are mutually shared to create collagen destruction and Hyp release into both saliva and plasma.

## 1 INTRODUCTION

Many studies have been reported in which diseases of the periodontium appear to be associated with systemic disease conditions.^1^ In this study of children, various metabolites in saliva and plasma were identified and their relationship to different levels of obesity and gingivitis was determined. Metabolomic molecules are the small molecules, usually < 1.5 KDa^2^ that provide a fingerprint of preceding molecular events. The objective of this approach is to identify pathways that are shared by both disease conditions as a fundamental basis for understanding their potential interaction.

Gingivitis and obesity have similarities yet are quite different pathologies. Gingivitis is an inflammatory disease of the mucosa surrounding the teeth that is of bacterial origin.^3^ Its inflammatory origins are distinct because gingival redness is visible and bacterial association is commonly reported. The natural reservoir for biochemical signals derived from gingivitis is saliva because inflammation is in the tissues surrounding teeth that are continuously bathed by saliva.

Obesity is an inflammatory disease of adipose tissue throughout the body created by excessive calorie consumption and metabolic disruption. C‐reactive protein (CRP) is produced by the liver, released (with other acute‐phase proteins) into the circulation, and is a marker of systemic‐inflammation. The inflammatory origins of obesity are not as apparent as those of gingivitis, but the release of CRP^4^ clearly points to an inflammatory component. The natural reservoir for biochemical signals derived from obesity is plasma. For these reasons, this study focused on the biochemicals that significantly correlated between saliva and plasma.

This communication summarizes findings that suggest that diseases so divergent in etiology as obesity and gingivitis may interact in ways that augment each other, a process being called disease reciprocity.

## 2 MATERIALS AND METHODS

### 2.1 Patients

Whole saliva and plasma samples were obtained from 68 children of both sexes (43 boys and 25 girls) between 7 and 15 years of age. Subjects were recruited by advertisements both in Cambridge, Massachusetts and Portland, Maine from February 2011 to September 2011. This study was approved by the human subjects ethics board the Forsyth Institutional Review Board and was conducted in accordance with the Helsinki Declaration of 1975, as revised in 2013. Both parental informed consent and the child's assent were obtained before their enrollment. The consent to publish results of this study were obtained as part of the informed consent procedure. These were written and are now stored as confidential filings.

### 2.2 Saliva and blood sample collection

In each case, saliva and blood samples were collected under fasting conditions (in the morning before breakfast or at least 3 hours after a meal). Five mL of blood and 3 mL saliva were collected from each. Each child rinsed their mouth with 15 mL of water and swallowed before saliva collection (not timed). Whole saliva (≈ 3 mL) was collected in labeled, sterile, 15‐mL plastic screw‐top centrifuge tubes (#430791, Corning Incorporated Life Sciences, Tewksbury, Massachusetts, USA) by unstimulated drooling using standard techniques.^5^

Saliva samples maintained on ice were rapidly transported to a laboratory where they were centrifuged at 2800 RPM for 20 minutes to remove the particulate debris and exfoliated mucosal cells. Supernatant saliva was transferred to screw‐cap, 1‐mL, 2D‐barcoded storage tubes (Matrix 2D Barcoded ScrewTop storage tubes, Thermo Fisher Scientific Inc., Hudson, New Hampshire, USA) and read by a barcode reader (Thermo Scientific VisionMate ST Barcode Reader, Thermo Fisher Scientific Inc.), by which participant number was related to sample number.

Immediately following saliva collection, blood was taken by a phlebotomist from the median cubital artery (BD Hemogard plus, K2 BD367863, lavender cap), centrifuged 10 minutes at 2000 × *g* in a refrigerated centrifuge. Both saliva and plasma samples were stored at −80°C until assayed.

### 2.3 Clinical assessments

Clinical evaluations were conducted by a trained examiner. Weight was measured by a calibrated bathroom scale, and waist circumference was measured by tape. Blood pressure and heart rate were measured by an automated cuff reading after the children had sat quietly for 10 minutes. Dental measurements were obtained using a portable pediatric dental chair, intraoral light, and mouth mirror. Gingivitis was evaluated by counting the number of red gingival sites (mesial, buccal, distal, and lingual of each tooth) by the primary clinical examiner. Dental caries was determined by counting the number of teeth with fillings or unfilled cavities. Neither dental probes nor radiographs were used. Fitness was measured by heart rate increase (beats/min) following a standard 3‐minute exercise using the Queens College step test^6^ and a finger‐tip heart rate monitor.

### 2.4 Metabolomic analysis

Aliquots of saliva supernatants and of plasma samples (120 µL) from each participant were assayed. Relative amounts of each metabolite were obtained by integrating peaks detected on an untargeted metabolic profiling platform (Metabolic, Durham, North Carolina, USA) employing high‐performance liquid chromatography/tandem mass spectrometry (HPLC‐MS/MS and gas chromatography‐mass spectrometry (GC‐MS) for volatile species.^7^ Compounds were identified by matching chromatographic retention times and mass spectral fragmentation signatures with reference library data created from authentic standards. By this method, the amino acid hydroxyproline was identified by having both the correct elution position by HPLC and the correct molecular weight by mass spectroscopy.

### 2.5 Data analysis

Analysis of saliva samples by untargeted metabolomics identified 245 biochemicals and analysis of plasma samples from the same children identified 336. To investigate the relationship between the body compartment and the oral cavity, 29 biochemicals that exhibited a significant correlation (*P* ≤ 0.01) were studied (Table S1 in online *Journal of Periodontology*).

From these 29 samples, 16 were excluded as being primarily as being xenobiotics or strongly related to food consumption. The remaining 13 biochemicals were investigated for their potential association between obesity and gingivitis disease conditions.

At the primary level, an analysis was conducted to determine the correlation between saliva levels and plasma levels, identifying those biochemicals in saliva that may be related to plasma. This analysis, if positive and significant, would indicate whether a positive distribution coefficient between the plasma and whole saliva compartments exists. If negative or insignificant, there is likely no relationship between saliva and plasma levels so that these variables are unlikely surrogate variable candidates. This assumes, however, that the concentration in both saliva and plasma is within the measurement sensitivity of the mass spectrometric method.

Obesity was evaluated by waist circumference using criteria suggested by the International diabetes foundation.^8^ This identified children equaling or exceeding the 90^th^ percentile of 10‐year‐old children. Gingivitis was based on the percentage of gingival sites considered red rather than pink. For analysis, gingivitis was considered to include the children with greater than or equal to the median value of 10.2% of gingival sites exhibiting erythema. Analysis of disease association was by *t*‐test using our definitions of obesity and gingivitis as the grouping variable and the total ion count of each biochemical as the selected variable. This created four analytical cases; obesity biomarkers in plasma, gingivitis biomarkers in saliva, gingivitis biomarkers in plasma and obesity biomarkers in saliva.

## 3 RESULTS

Overall statistics (Table 1) demonstrate that the average age was 10.67 years, and 43 of the children (63.2%) were male. In this study, sex was not identified as an important covariate. Although the prevalence of obesity in boys (39.5%) was approximately twice that of girls (20%), boys had only 1.06 more permanent teeth, and 4.75% more red sites than girls neither of which were statistically significant Chi‐square analysis of both obesity and gingivitis with sex in Table 1 are also not statistically significant. Statistics based on obesity (90th percentile) of these children demonstrate that obese children had higher BMI (30.0 versus 18.4 kg/m^2^), greater waist circumference (96.4 versus 63.6 cm) and reduced physical fitness (40.9 versus 23.8 beats/min), consistent with recognized characteristics of obesity. The average weight of obese children was twice that of not obese children (76.6 versus 38.5 Kg). Systolic blood pressure was higher in obese children but of borderline significance (*P* = 0.06). Obese children had 5.6 more permanent teeth (21.1 versus 15.5) and five fewer deciduous teeth (2.6 versus 7.6) indicating that obese children had premature tooth eruption. Gingival sites in obese children had a significantly higher percentage of red sites (30.l8% compared to 10.2%) indicating that gingivitis was significantly elevated in obese children.

**TABLE 1:** Characteristics of 68 children participating in this study demonstrating measures associated with obesity (by waist circumference) and gingivitis (by percent red gingival sites)

	**Obesity statistics**			**Gingivitis statistics**			
**Parameter**	**Not obese**	**Obese**	***P* (Obese)**	**Not gingivitis**	**Gingivitis**	***P* (Gingivitis)**	**Overall**
*N*	46	22		34	34		68
Age (years)	10.1	11.8		10.43	10.90		10.67
Male (*n*)	26	17		23	20		43
Male (%)	56.5	77.3	0.17	67.6	58.8	1.00	63.2
Permanent teeth (*N*)	15.5	21.1	**0.0001**	16.7	18.5	0.23	17.6
Deciduous teeth (*N*)	7.6	2.6	**0.00006**	6.6	5.6	0.40	6.1
Height (cm)	143.4	157.8	**0.00001**	146.2	150.0	0.25	148.1
Weight (kg)	38.5	76.6	**≤0.00001**	43.7	57.9	**0.02**	50.8
BMI (kg/M^2^)	18.4	30.0	**≤0.00001**	19.9	24.4	**0.009**	22.2
Waist circumference (cm)	63.6	96.4	**≤0.00001**	68.4	80.2	**0.01**	74.4
Fitness (beats/min)	23.8	40.9	**0.0004**	26.9	31.8	0.30	29.4
Systolic BP (mmHg)	118.9	124.0	0.06	122.25	118.82	0.19	120.54
Diastolic BP (mmHg)	67.7	70.1	0.20	69.82	67.16	0.13	120.54
Heart rate (beats/min)	79.2	77.9	0.71	78.00	79.56	0.63	68.49
Decayed or filled (%)	5.3	5.3	0.99	5.69	4.91	0.71	5.30
Red gingival sites (%)	10.2	30.8	**≤0.00001**	4.1	28.8	**≤0.00001**	16.6

In statistics based on gingivitis (% of sites with gingival redness ≥ 10.6%) children with gingivitis also had significantly higher weight (57.9 versus 43.7 Kg), higher body mass index (BMI) (24.4 versus 19.9 Kg/m^2^) and greater waist circumference (80.2 versus 68.4 cm) indicating that children with gingivitis were significantly associated with obesity. In addition to the expected association with gingival redness.

Dental decay, though present, was not related to either obesity or gingivitis. Also, diastolic blood pressure and heart rate were not associated with obesity or gingivitis.

Metabolomic analysis of 29 biochemicals found to be significantly correlated between plasma and saliva (Table 2 and Table S2 in online *Journal of Periodontology*) were further separated into undefined biochemicals and food or xenobiotic related biochemicals. This analysis focused on those undefined biochemicals that are potentially disease‐related, excluding those that are food‐related or xenobiotics.

**TABLE 2:** Systemic metabolites found to significantly correlate between plasma and saliva

**Saliva**		
**Metabolites**	***P* (Gingivitis)**	**Biomarker status**
trans‐4‐Hydroxyproline	**0.01**	Collagen breakdown
Creatinine	**0.05**	Kidney disease?
Urate	0.09	Fructose consumption
N1‐Methyl‐2‐pyridone‐5‐carboxamide	0.10	Nicotinate and nicotinamide metabolism
Cortisone	0.10	Secreted in response to stress
3‐Hydroxybutyrate (BHBA)	0.16	Ketone bodies
2‐Hydroxybutyrate (AHB)	0.17	Cysteine, methionine, SAM, taurine metabolism
1,5‐Anhydroglucitol (1,5‐AG)	0.17	Glycolysis, gluconeogenesis, pyruvate metabolism
1,2‐Propanediol	0.19	Ketone bodies
Betaine	0.20	Glycine, serine and threonine metabolism
Isovalerylcarnitine	0.56	Valine, leucine and isoleucine metabolism
3‐Indoxyl sulfate	0.61	Tryptophan metabolism
Fructose	0.85	Starch, and sucrose metabolism
Propionylcarnitine	0.91	Fatty acid metabolism
2‐Methylbutyroylcarnitine	0.91	Valine, leucine and isoleucine metabolism

**Plasma**		
**Metabolites**	***P* (Obesity)**	**Biomarker status**
Creatinine	**0.0001**	Kidney disease?
Urate	**0.0002**	Fructose consumption
Cortisone	**0.004**	Secreted in response to stress
3‐Hydroxybutyrate (BHBA)	**0.03**	Ketone bodies
isovalerylcarnitine	0.08	Valine, leucine and isoleucine metabolism
2‐Methylbutyroylcarnitine	0.09	Valine, leucine and isoleucine metabolism
1,2‐Propanediol	0.09	Ketone bodies
3‐Indoxyl sulfate	0.13	Tryptophan metabolism
trans‐4‐Hydroxyproline	0.25	collagen breakdown
1,5‐Anhydroglucitol (1,5‐AG)	0.32	Glycolysis, gluconeogenesis, pyruvate metabolism
Fructose	0.44	Starch, and sucrose metabolism
Propionylcarnitine	0.50	Fatty acid metabolism
Betaine	0.79	Glycine, serine and threonine metabolism
2‐Hydroxybutyrate (AHB)	0.83	Cysteine, methionine, SAM, taurine metabolism
N1‐Methyl‐2‐pyridone‐5‐carboxamide	.97	Nicotinate and nicotinamide metabolism

The calculated probability (*P*) identifies hydroxyproline as a principle metabolomic biomarker for identification of the % of red gingival sites (gingivitis).

Hydroxyproline (Hyp) was found to be a major metabolite (*P* = 0.01) associated with gingivitis in saliva (Table 2). HYP also appeared in plasma with a non‐significant (*P* = 0.25) relationship to obesity. The plasma: saliva correlation coefficient for Hyp was 0.37 with *P* = 0.002 (Table S1 in online *Journal of Periodontology*).

**FIGURE 1:**
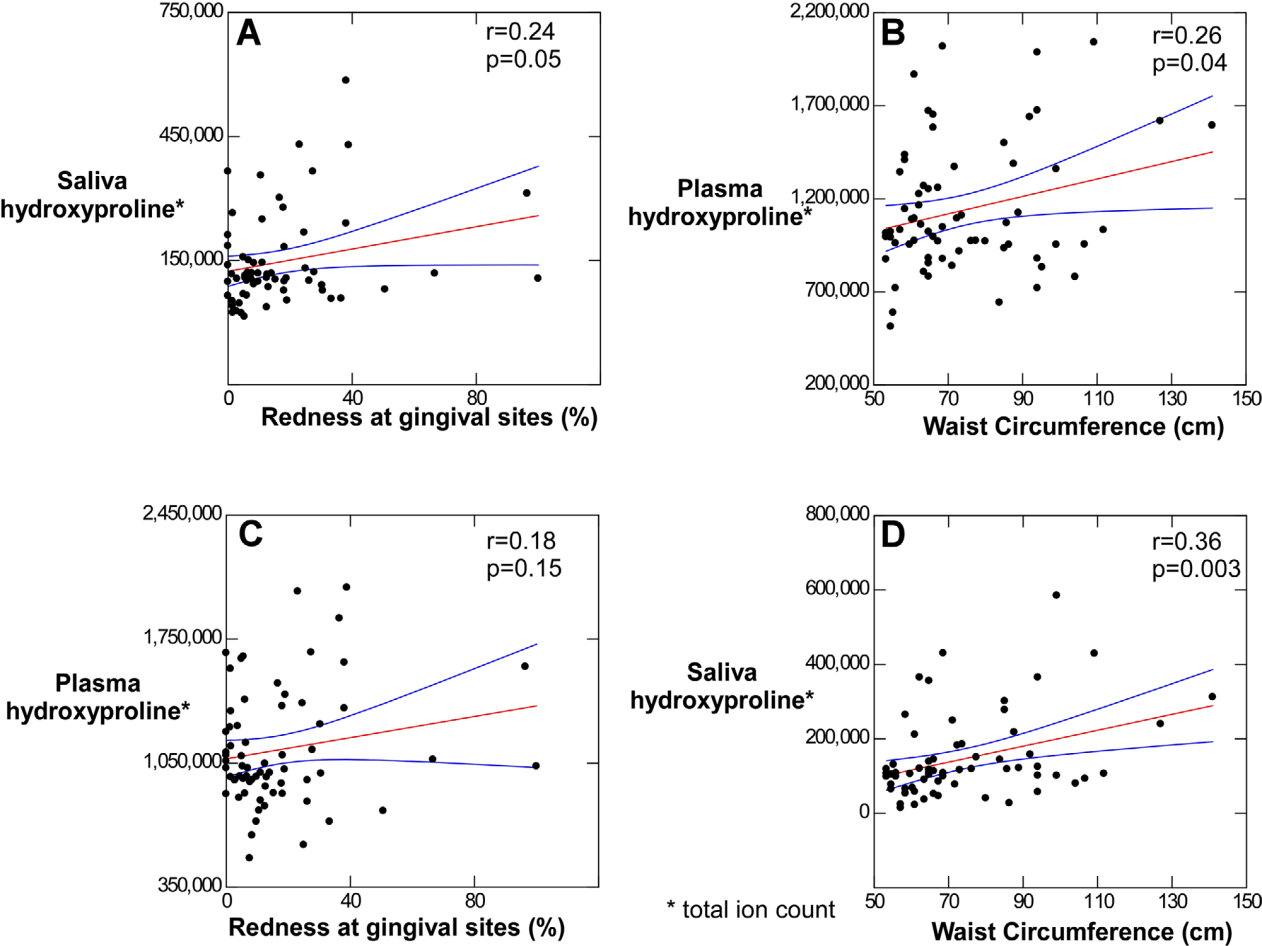
By regression, both obesity and gingivitis produce hydroxyproline and augment each other

Regression analysis of the total ion count of Hyp in plasma and in saliva related to waist circumference and gingival redness (Figure 1) indicating a positive slope in all cases. All relationships were significant except for the presence of Hyp in plasma associated with gingivitis (Figure 1C).

It should be noted that regression between obesity measured by waist circumference and gingivitis measured by percent of red sites had a high correlation (r = 0.66) and was highly significant (*P* = 0.00002).

**FIGURE 2:**
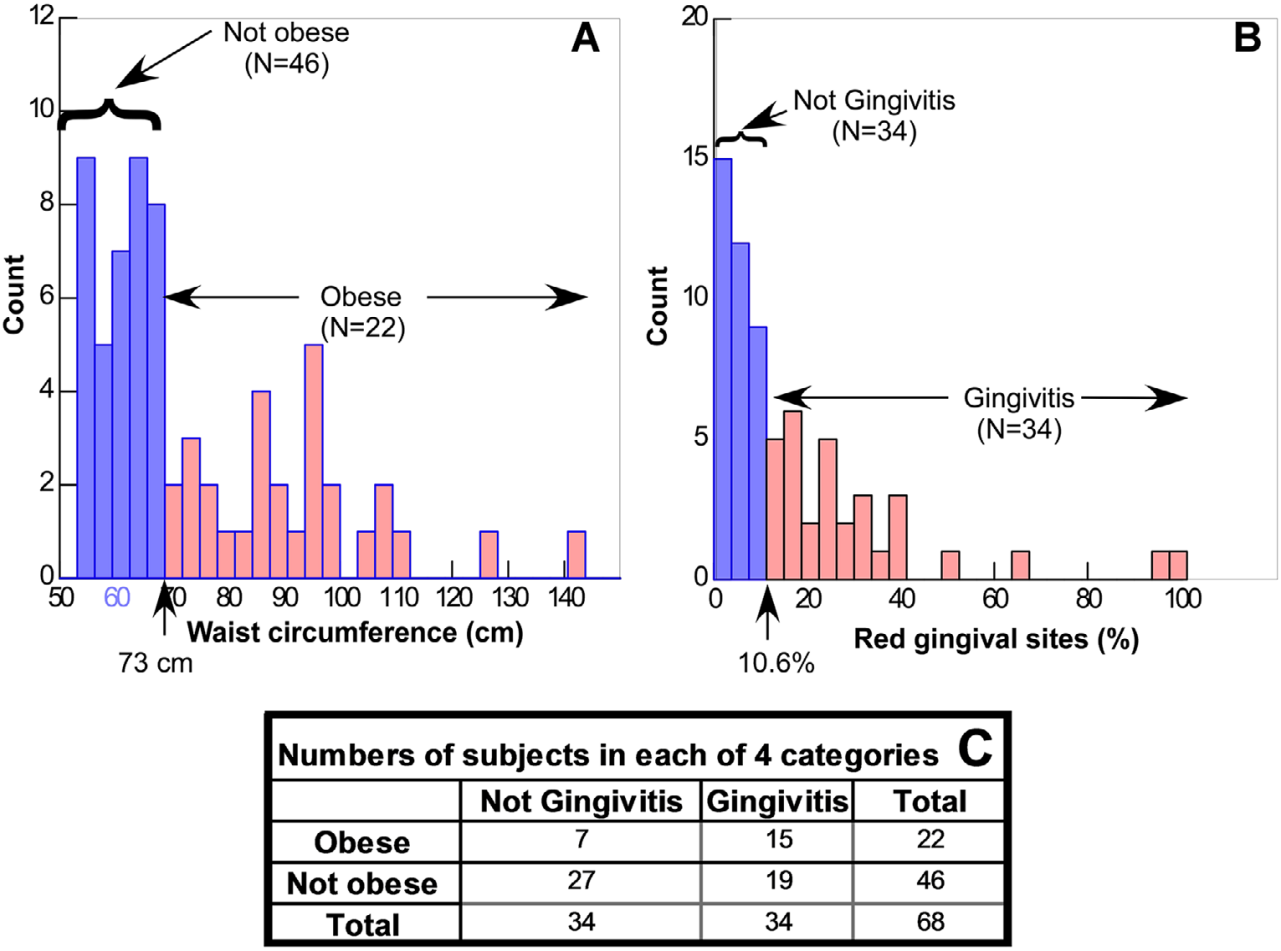
Distribution of waist circumference and % of red gingival sites. Obese children (**A**) were identified by their waist circumference being ≥ 90^th^ percentile of European children (73 cm). Children with gingivitis were identified by the percentage of red gingival sites (**B**) that exceeded the median value (10.6%). Numbers of subjects in each of the defined four categories (**C**) varied from seven (not gingivitis but obese) to 27 (not gingivitis and not obese)

To illustrate Hyp distribution among subjects, gingivitis was defined as those children with the median value ≥10.6% of gingival sites being red (Figure 2B) and obesity was defined as those children who had waist circumference exceeding the 90th percentile of 10‐year‐old children (Figure 2A). This characterization of the population defines four groups (Figure 2C); those with no gingivitis or obesity (n = 27), those not obese with gingivitis (n = 19), those without gingivitis and with obesity (N = 7) and those with both obesity and gingivitis (N = 15).

**FIGURE 3:**
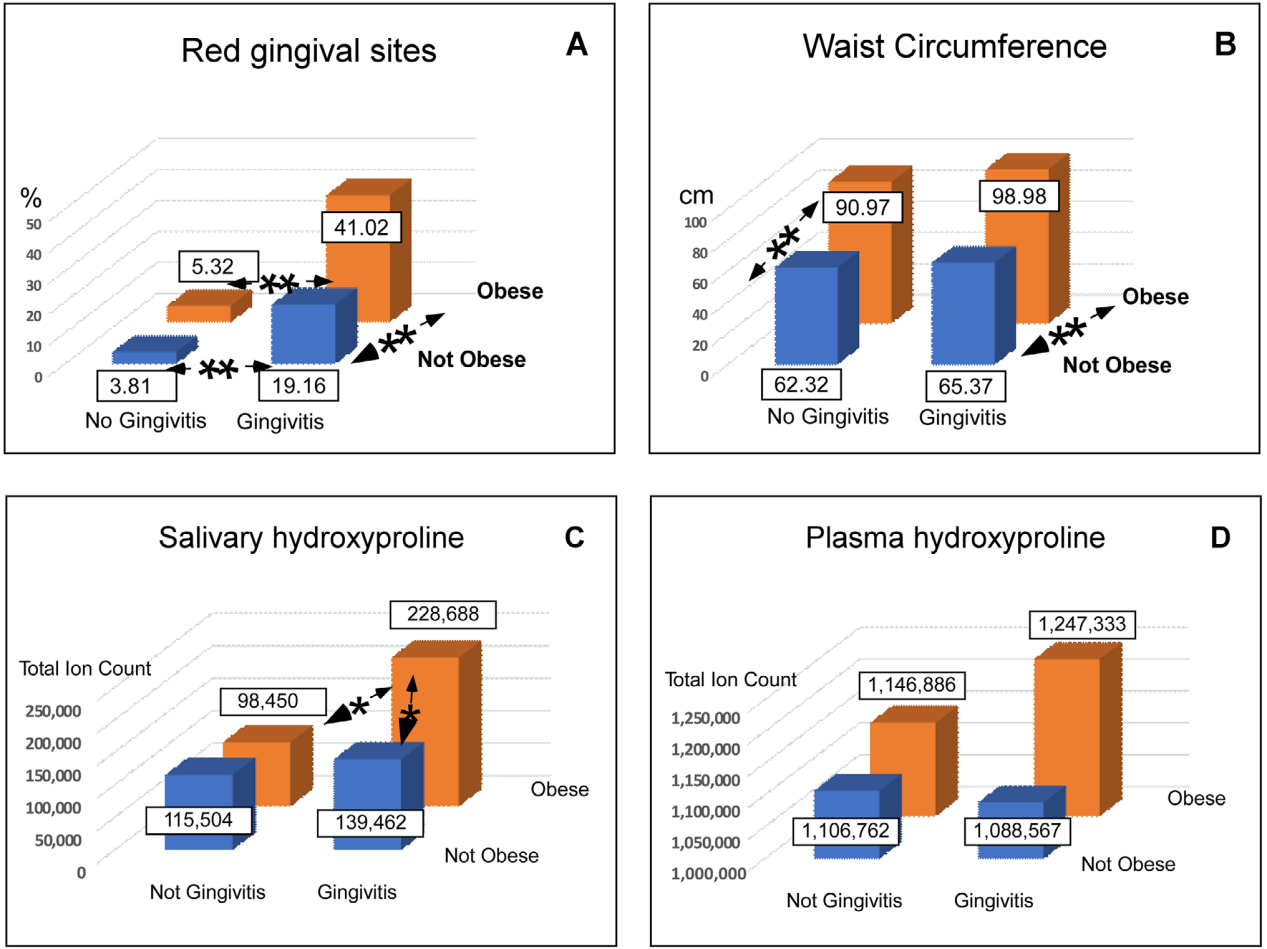
Gingival site redness percent (**A**), waist circumference in centimeters (**B**), salivary hydroxyproline (**C**) and plasma hydroxyproline (**D**) related to obesity and gingivitis. In children with no gingivitis, gingival site redness was not significantly affected by obesity. If children had gingivitis and were obese, however, the red gingival site % was doubled (A). Waist circumference was greater in children with gingivitis, but this difference was not statistically significant (B). Salivary hydroxyproline (Hyp) (C) was significantly increased by gingivitis with obesity. Plasma Hyp (D) was also increased by the presence of both gingivitis and obesity, but this difference was not statistically significant. *P*‐values marked by ** were < 0.001 and *P*‐values marked by * were < 0.05

Using this definition of gingivitis, one can view the effects as a 3D column distribution (Figure 3A). Considering the percent of red gingival sites, the presence of both gingivitis and obesity approximately doubled the extent of gingival redness (19.16% to 41.02%). If there was no gingivitis, however, obesity had little effect on the % of red gingival sites (3.81% to 5.32%). Waist circumference (Figure 3B) was increased by the presence of gingivitis (90.97 to 98.98), which was not statistically significant (*P* = 0.3).

Parallel to these clinical observations, the amount of salivary and plasma Hyp changed similarly. In saliva (Figure 3C), without gingivitis there was little difference in Hyp between obese and not obese children (*P* = 0.6). If children were both obese and had gingivitis, however, there was a major difference in Hyp (a total ion count of 139,462 to 228,688, *P* = 0.05). In plasma (Figure 3D), a similar pattern was seen except that without gingivitis there was a small increase in Hyp (total ion count of 1,106,762 to 1,146,886) but little change in Hyp with gingivitis in the absence of obesity. Plasma values were like those of saliva, but they were not statistically significant.

## 4 DISCUSSION

It seems that these two diseases, obesity and gingivitis support each other. If one is obese, their gingivitis is amplified. If one has gingivitis, the pathway to obesity is facilitated.

Hyp, has long been considered a biomarker for collagen destruction.^9^ Collagen contains ≈ 13.5% Hyp^10^ and is found in few other proteins. The destruction of collagen is a stepwise process by which collagen bundles are eventually degraded to peptides and amino acids. Hyp, formed as a post‐translational modification of proline, is not reused for collagen synthesis but is generally excreted.^11^

Hyp is associated with both gingivitis and periodontitis,^12^ where it is associated with activated neutrophil matrix metallopeptidase 8 (MMP8). The process of neutrophil activation is prominent it these disease conditions.^13^ Although circulating neutrophils seem the likely candidate the release of HYP under the conditions studied, it should be recognized that macrophages, fibroblasts, and some epithelial cells also possess a collagenase.^14^

Obesity is also associated with collagen destruction. This is clearly documented in this study by increased skeletal changes (Table 1). The height of obese children was significantly greater than those not obese (157.8‐143.4 = 14.4 cm). The increased number of permanent teeth (21.1‐15.5 = 5.6 teeth) and the decreased number of primary teeth (7.6‐2.5 = 5.1 teeth) indicating increased tooth eruption rate. Both differences in skeletal metabolism require increased collagen destruction.

The association between increased gingivitis and obesity in children has been reported by others.^15^ The current study also points to evidence of collagen destruction by the appearance of Hyp in saliva increasing with increased gingival redness (Figure 1A) and increased Hyp in plasma associated with increased waist circumference (Figure 1B).

In this study, a significant (*P* ≤ 0.00001) association between obesity and red gingival sites was indicated by obese children having 30.8% redness compared to 10.2% sites in the absence of obesity (Table 1). Also, the presence of obesity significantly (*P* = 0.003) increased Hyp in saliva (Figure 1D). The effect of obesity on gingivitis is illustrated in Figure 3A where 41.02% of sites in subjects defined as having both gingivitis and obesity exhibited redness compared to 10.2% redness for obesity in the absence of gingivitis and 19.6% redness for gingivitis in the absence of obesity. Salivary Hyp was significantly greater in children defined as having gingivitis plus obesity than those with gingivitis alone and obesity alone (Figure 3C). The impact of obesity on gingivitis also suggests why so few subjects (*N* = 7) were categorized as having obesity with no gingivitis (Figure 2C).

The converse, an effect of gingivitis on obesity, is implied but not proven by this relatively small study. Several measures of obesity (height, weight, and waist circumference) were significantly associated with gingivitis (Table 1). In the presence of obesity, plasma Hyp increased (total ion count of 1,146,886 to 1,247,333) with gingival redness (Figure 3D), but this was not statistically significant (*P* = 0.15). The difference in waist circumference between children with gingivitis alone and gingivitis with obesity (65.37 versus 98.98 cm) was significant (*P* ≤ 0.001), but the difference between obesity alone and obesity with gingivitis (90.97 versus 98.98) was not. Although the pattern in plasma Hyp (Figure 3D) was similar to that of saliva Hyp (Figure 3C), none of the differences in plasma were statistically significant. One explanation is for the relatively small effect of gingivitis on obesity is dilution. The tissue mass associated with gingivitis being much smaller than that of obesity. Obese children in this study weighed twice as much as those who were not obese (76.6 versus 38.5 kg), a value many times greater than that of tissue afflicted with gingivitis.

**FIGURE 4:**
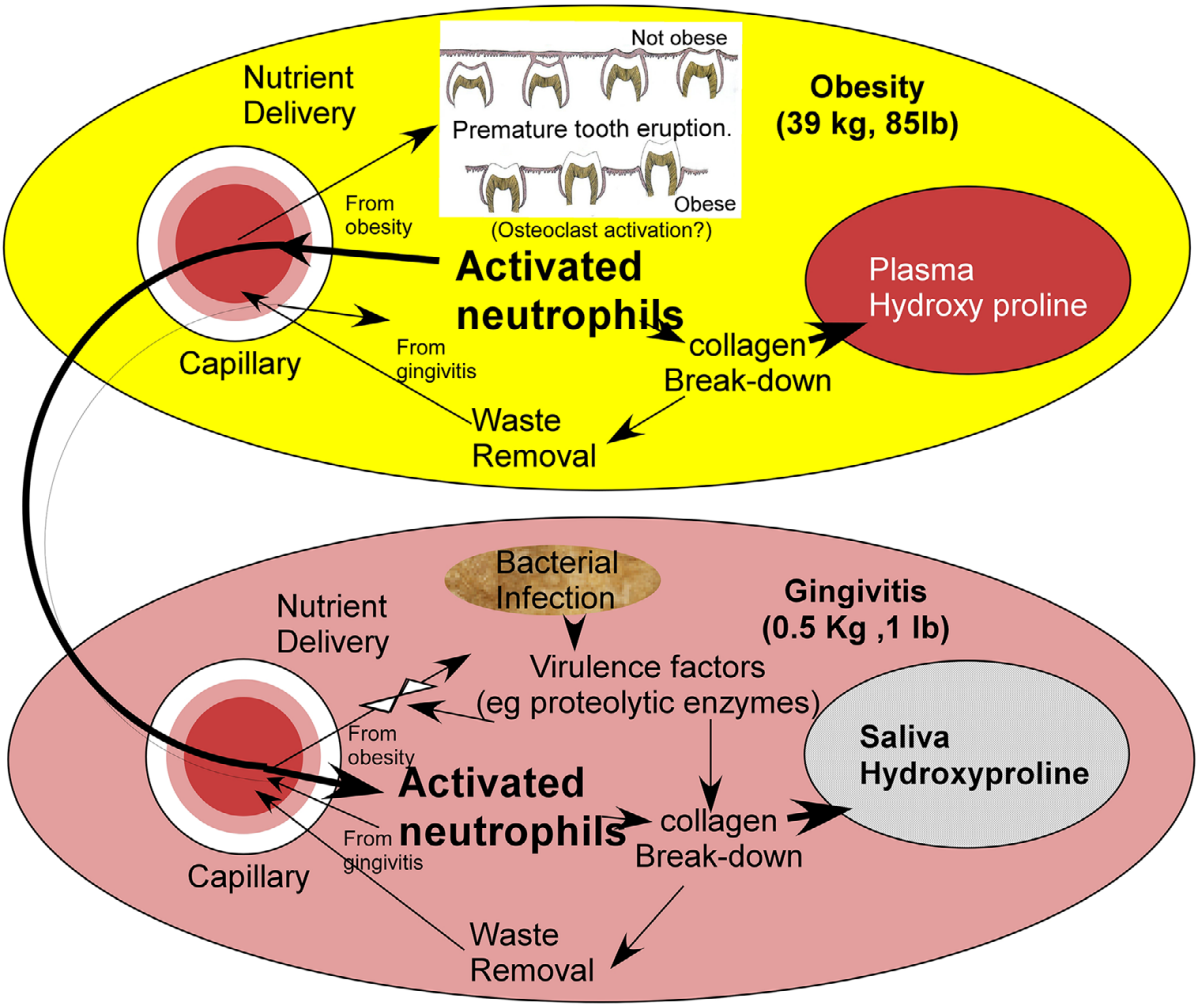
Disease reciprocity suggests that activated neutrophils are used by both gingivitis and obesity to digest collagen resulting in hydroxyproline release into saliva and plasma. The thick arrow from obesity to gingivitis and the thin arrow from gingivitis to obesity reflect the larger tissue involvement in obesity

These observations lead one to a consideration of disease reciprocity (Figure 4). Reciprocity is the practice of exchanging things for mutual benefit. The concept that diseases can work together is suggested in this juxtaposition of obesity with gingivitis. Two different diseases, both engaged with collagen breakdown showing signs of mutual augmentation. With tooth eruption, an obesity stimulated reaction, one expects osteoclastic action and inflammation with neutrophil infiltration as the tooth breaks the oral mucosa and erupts into the mouth. Hence, tooth eruption would be expected to involve neutrophil activation and collagen degradation leading to Hyp release into the plasma. With gingivitis, bacterial presence around the teeth results in neutrophil localization and activation and inflammation, which ultimately creates collagen degradation leading to Hyp release into saliva. Because both diseases involve neutrophil activation, they may well mutually benefit as diseases by receipt of neutrophils activated by the other. These two diseases exhibit reciprocity. The potential size of obesity‐controlled tissue being much larger than the oral environment so that the effect of gingivitis on obesity is largely diluted so that gingivitis has only a small degree of influence on obesity; however, the impact of obesity on gingivitis is major. To encourage alternative analyses by interested readers, the data and variable descriptions for this study (Table S3 in online *Journal of Periodontology*).

The presence of high levels of creatinine and uric acid (Table 2) in the plasma and saliva of obese children suggests the possibility of association with kidney disease. Chronic kidney disease has also been associated with periodontitis.^16^

## 5 CONCLUSIONS

This study suggests that obesity enhances gingivitis and gingivitis enhances obesity through a mechanism involving neutrophil activation and resulting in hydroxyproline release by collagen degradation. It is proposed that a process of disease reciprocity is occurring in which these diseases aid each other. These results explain how multiple diseases can be overwhelming and how effective therapy for one disease can relieve the effects of another.

## Supporting information

Supplemental Table 1

Supplemental Table 2

Supporting Information
