# Supplementary material for: Disease reciprocity between gingivitis and obesity
Source: J Periodontol. 2020 Aug 6;91(Suppl 1):S26–34. doi: 10.1002/JPER.20-0046 (PMC7689803; doi:10.1002/JPER.20-0046)
Supplement: Supplementary file 1 — Supplemental Table 1 [file JPER-91-S26-s001.docx]

Supplemental Table 1, Number of subjects with biochemicals in saliva and plasma, along with correlation "r" and the p-value of this correlation.

| **BIOCHEMICAL** | **Nsaliva** | **Nplasma** | **r** | **p** |
| --- | --- | --- | --- | --- |
| creatinine | 68 | 68 | 0.62 | 0.00000002 |
| 1,2-propanediol | 68 | 68 | 0.98 | 0.00000002 |
| phenol sulfate | 68 | 68 | 0.74 | 0.00000002 |
| stachydrine | 64 | 68 | 0.90 | 0.00000002 |
| hippurate | 54 | 68 | 0.94 | 0.00000002 |
| tryptophan betaine | 36 | 66 | 0.86 | 0.00000002 |
| catechol sulfate | 64 | 68 | 0.91 | 0.00000002 |
| theobromine | 62 | 64 | 0.94 | 0.00000002 |
| fructose | 65 | 68 | 0.74 | 0.00000002 |
| 3-indoxyl sulfate | 56 | 68 | 0.77 | 0.00000002 |
| caffeine | 47 | 65 | 0.76 | 0.00000002 |
| 3-hydroxybutyrate (BHBA) | 62 | 68 | 0.63 | 0.00000005 |
| p-cresol sulfate | 64 | 68 | 0.62 | 0.00000006 |
| paraxanthine | 48 | 24 | 0.85 | 0.00000012 |
| urate | 68 | 68 | 0.58 | 0.0000003 |
| pipecolate | 67 | 68 | 0.48 | 0.00003 |
| cortisone | 61 | 68 | 0.49 | 0.00006 |
| N1-Methyl-2-pyridone-5-carboxamide | 67 | 55 | 0.49 | 0.0001 |
| 1,5-anhydroglucitol (1,5-AG) | 68 | 68 | 0.44 | 0.0001 |
| isovalerylcarnitine | 66 | 68 | 0.45 | 0.0002 |
| 2-methylbutyroylcarnitine | 45 | 68 | 0.51 | 0.0003 |
| erythritol | 49 | 68 | 0.44 | 0.001 |
| propionylcarnitine | 68 | 68 | 0.38 | 0.002 |
| 2-hydroxybutyrate (AHB) | 66 | 68 | 0.38 | 0.002 |
| trans-4-hydroxyproline | 65 | 68 | 0.37 | 0.002 |
| theophylline | 19 | 44 | 0.68 | 0.003 |
| carnitine | 68 | 68 | 0.35 | 0.004 |
| betaine | 68 | 68 | 0.34 | 0.004 |
| caproate (6:0) | 42 | 68 | 0.39 | 0.01 |
