# Supplementary material for: Disease reciprocity between gingivitis and obesity
Source: J Periodontol. 2020 Aug 6;91(Suppl 1):S26–34. doi: 10.1002/JPER.20-0046 (PMC7689803; doi:10.1002/JPER.20-0046)
Supplement: Supplementary file 2 — Supplemental Table 2 [file JPER-91-S26-s002.docx]

Supplemental Table 2. Food related and Xenobiotic metabolites found to significantly correlate between plasma and saliva.

| **Saliva** | | |
| --- | --- | --- |
|  |  |  |
| **Food-related and Xenobiotics** | **p(Gingivitis)** | **Biomarker status** |
| pipecolate | **0.02** | dried cooked beans |
| phenol sulfate | **0.05** | many fruits and vegtables |
| carnitine | **0.05** | meat |
| theobromine | **0.05** | chocolate |
| caproate (6:0) | 0.10 | Animal Fat |
| theophylline | 0.10 | Tea |
| erythritol | 0.11 | pear, grapes |
| paraxanthine | 0.21 | coffee |
| caffeine | 0.26 | coffee |
| catechol sulfate | 0.36 | papaya |
| stachydrine | 0.44 | citrus |
| tryptophan betaine | 0.61 | lentil |
| hippurate | 0.64 | tea, wine and fruit juices |
| p-cresol sulfate | 0.96 | microbial metabolite |
| **Plasma** | | |
|  |  |  |
| **Food-related and Xenobiotics** | **p(Obesity)** | **Biomarker status** |
| caproate (6:0) | **0.002** | Animal Fat |
| erythritol | **0.03** | pear, grapes |
| carnitine | **0.04** | meat |
| p-cresol sulfate | **0.04** | microbial metabolite |
| paraxanthine | 0.14 | coffee |
| theophylline | 0.17 | Tea |
| theobromine | 0.18 | chocolate |
| caffeine | 0.20 | coffee |
| stachydrine | 0.24 | citrus |
| pipecolate | 0.56 | dried cooked beans |
| hippurate | 0.64 | tea, wine and fruit juices |
| tryptophan betaine | 0.85 | lentil |
| phenol sulfate | 0.94 | many fruits and vegtables |
| catechol sulfate | 0.97 | papaya |
